# Bioactive Compounds from Dandelion (*Taraxacum officinale*): Advances in Extraction Techniques and Applications

**DOI:** 10.3390/foods15040782

**Published:** 2026-02-21

**Authors:** Lynn Rhayem, Nadia Boussetta, Mirian T. K. Kubo, Franck Merlier, Eugene Vorobiev, Nikolai Lebovka

**Affiliations:** 1 Ecole Supérieure de Chimie Organique et Minérale, Alliance Sorbonne Université, TIMR, Université de Technologie de Compiègne, 60200 Compiègne, France; lynn.rhayem@utc.fr (L.R.); eugene.vorobiev@utc.fr (E.V.); 2 CNRS, Génie Enzymatique et Cellulaire, Centre de Recherche Royallieu, Université de Technologie de Compiègne, UPJV, 60200 Compiègne, France; mirian.kubo@utc.fr (M.T.K.K.); franck.merlier@utc.fr (F.M.); 3 F. D. Ovcharenko Institute of Biocolloidal Chemistry, Department of Physical Chemistry of Disperse Minerals, National Academy of Sciences of Ukraine, 42, Akademika Vernadskoho boulevard, 03142 Kyiv, Ukraine; lebovka@gmail.com

**Keywords:** dandelion, extraction, bioactive compounds, conventional and innovative extraction techniques

## Abstract

*Taraxacum* is a genus of flowering plants comprising species commonly known as dandelions. All parts of the dandelion (flowers, stems, roots, and leaves) contain valuable bioactive compounds, including flavonoids, amino, fatty, organic, and phenolic acids, coumarins, lignans, polysaccharides, phytosterols, terpenes, glycoproteins, oligosaccharides, and alkaloids. Dandelion extracts represent a promising feedstock for diverse applications across the food, biomedical, and pharmaceutical industries. The extraction of bioactive compounds from dandelion is essential to access its therapeutic properties, with different techniques used to isolate its various phytochemicals. This review provides a comprehensive overview of recent advances in the application of various techniques for the extraction of bioactive compounds from dandelion. Both conventional and innovative extraction techniques are discussed, with particular emphasis on their respective advantages and limitations.

## 1. Introduction

Dandelion (*Taraxacum officinale)* is a wild species with remarkable nutritional and medicinal properties. It is an herbaceous perennial flowering spring plant that belongs to the Asteraceae family. It is part of the Cichorioideae subfamily [1] and the Lactuceae tribe [2] and is classified under the genus *Taraxacum*. The genus name *Taraxacum* is derived from the Greek words *taraxos* (disorder) and *akos* (remedy), reflecting its traditional use in treating ailments. The specific epithet *officinale* indicates that the plant possesses medicinal properties [3]. The dandelion belongs to the family Asteraceae and it includes more than 22,000 species [4]. Some botanists classify this genus into 60 macrospecies, while others divide it into 2000 microspecies belonging to 34 sections, also interpreted as macrospecies [5].

Dandelion is widely distributed across hillside grasslands, roadsides, fields, and riverbanks in low- and mid-altitude regions. It occurs naturally in Europe, Asia, and North America. Studies conducted in various geographical regions have shown that the chemical composition of dandelion varies depending on environmental factors such as soil quality, climate, and harvest time [6]. Dandelion germinates early in spring, exhibiting rapid growth at temperatures around 8–10 °C. The flowering period generally extends from mid- to late May and lasts for approximately 40 days. Recent studies have examined the effects of varying seeding rates and harvest regimes on dandelion cultivation [7]. Different seeding rates and harvest regimes were compared, and the results demonstrated that neither factor had a significant effect on dandelion productivity. Moreover, the authors stated that high-density cultivation of dandelion showed promising performance, indicating potential for large-scale industrial purposes.

Dandelion plants possess a well-developed taproot system with secondary lateral roots, and their foliage is arranged in a basal rosette from which the flowering stem emerges. The species is a perennial herb that develops from a robust, fleshy taproot, dark brown on the exterior. Its long, green leaves form a basal rosette at ground level. The inflorescences (flower heads) vary in colour from yellow to orange, while the fruits are brown and bear a white, hairy pappus that enables wind dispersal.

Although dandelion is considered a weed, it is a widely consumed edible plant due to its rich composition of bioactive compounds, including amino acids, organic acids, carbohydrates, sesquiterpenoids, steroids and triterpenoids, fatty acids, caffeoylquinic acids, flavonoids, mineral salts, and vitamins [8].

Owing to its rich phytochemical composition, this plant exhibits a wide range of beneficial properties, including notable medicinal effects such as diuretic, hepatoprotective, anti-colitis, immunomodulatory, antiviral, antifungal, antibacterial, antidiabetic, anti-obesity, antioxidant, and anticancer activities [3]. Dandelion is utilized across various sectors, including food, medicine, and skincare, in forms such as raw plant material, extracts, herbal infusions, and powders. Numerous studies have examined the chemical composition of this plant and its relevance to human health [9].

Over the past decade, the extraction and quantification of bioactive compounds from various parts of the dandelion plant using both conventional and emerging techniques have been studied. Dandelion has been recognized as a promising source of functional ingredients, particularly for applications in the food industry. However, most of these studies have focused on specific applications of dandelion without sufficiently addressing its limitations and challenges. Moreover, recent developments in extraction methodologies and the analysis of its bioactive compounds—such as antioxidant activity, total phenolic content, and cytotoxicity—have yet to be comprehensively reviewed.

This article provides an overview of recent studies conducted over the past decade on the extraction of bioactive compounds from dandelion. It examines the plant’s life cycle, the chemical composition of its various parts, and its potential applications across multiple sectors, along with existing limitations. Furthermore, it explores the extraction and isolation of bioactive compounds, emphasizing the techniques, solvents, and analytical methods employed. Both conventional and emerging extraction technologies, such as instant controlled pressure drop, supercritical and subcritical fluid extraction, ultrasound-assisted extraction, microwave-assisted extraction, and electrically driven extraction methods, are comprehensively reviewed.

## 2. Life Cycle of Dandelion

**Figure 1:**
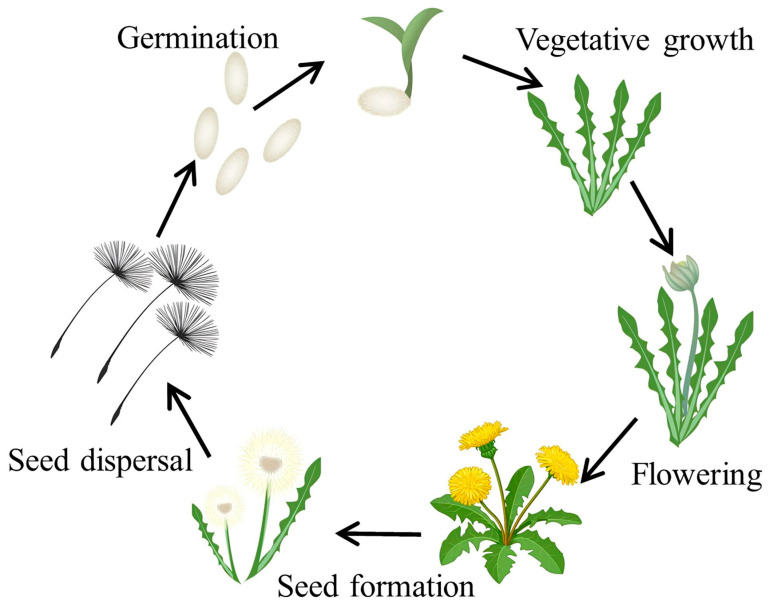
Dandelion life cycle.

The life cycle of the dandelion plant begins with seeds deposited on the soil surface. These seeds are dispersed by wind, and under favourable conditions, they germinate. The life cycle can be divided into four stages: germination, vegetative growth, flowering, and seed formation with subsequent seed dispersal (Figure 1).

During the germination step, a small root called a radicle grows from the seed downward into the soil. Germination is defined as the point at which the emerging radicle exceeds 1 mm in length [10]. Then, a shoot starts to grow upward, and emergence was recorded once the shoot became visible at or above the growth medium. This step typically lasts between 7 and 21 days. Afterwards, the dandelion enters the vegetative growth stage, during which true leaves start to develop. The leaves form a basal, radial rosette that stays close to the ground [11].

In the next stage of the life cycle, as the plant matures, the bright yellow flowers begin to bloom. This is the flowering stage. Five flowering phenology parameters were identified and monitored [12]. The initiation of the flowering process was first observed through the formation of an inflorescence bud at the centre of the basal rosette. Bud development was documented when a 5 mm bud segment could be seen at the centre of the rosette of leaves. After the formation of the bud, a hollow, leafless stem, known as the scape, begins to develop at the base. As elongation progresses, the inflorescence rises and opens into a flower head, that consists of several tightly packed yellow florets and is surrounded by two rows of green bracts. The inner bracts are typically larger and remain upright, while the smaller outer bracts reflex outward. Then, the seed formation stage follows the flowering stage.

Following the development of the flower head, the previously reflexed bracts contract as the inflorescence closes. During this process, the base of each floret dries and matures into a seed. Subsequently, the bracts retract to reveal a ‘white ball’ composed of numerous dry fruits (achenes), each attached to a parachute-like pappus that facilitates wind dispersal. The final stage of the dandelion life cycle is seed dispersal, during which the achenes detach and are carried by the wind to colonize new areas. Under favourable conditions, these seeds germinate to form new dandelion plants, thereby completing the life cycle. Remarkably, even after the removal of the aerial parts, the persistent taproots remain viable and can generate new plants. On average, each dandelion produces 5 to 10 inflorescences, each containing multiple achenes [13].

## 3. Chemical Composition

Detailed information on dandelion plant characterization, chemical structures of representative compounds, and bioactive compounds isolated from dandelion was recently discussed [14]. All parts of the dandelion (roots, leaves, flowers) are edible and contain bioactive compounds such as phenolic acids, alkaloids, flavonoids, and terpenes, with extracts from the leaves and roots being the most extensively studied. Dandelion contains a wide range of bioactive constituents, including flavonoids, amino acids, fatty acids, organic acids, phenolic acids, coumarins, lignans, polysaccharides, phytosterols, terpenes, glycoproteins, oligosaccharides, and alkaloids. Dandelion roots, leaves, and flowers contain phenolic acids and flavonoids as the principal classes of secondary metabolites, but each plant part shows a distinct distribution of compound subclasses. Roots are characterized by a higher abundance of hydroxybenzoic acids and flavonoid aglycones, with chlorogenic and caffeic acids also contributing to their phenolic profile. In contrast, leaves are dominated by hydroxycinnamic acid derivatives, particularly caffeoylquinic and chicoric acids, together with luteolin-based flavonoid glycosides. Flowers share many phenolic acids and flavonoid glycosides with leaves but are distinguished by the presence of chrysoeriol and a greater relative abundance of dicaffeoylquinic acids. The distribution of the major bioactive compounds found in each dandelion plant part is given in Table 1.

The quantification of phenolic compounds in an ethyl acetate extract of dandelion roots has been performed [15]. A total of 18 phenolics were identified by ultra-performance liquid chromatography-mass spectrometry (UPLC-MS), including apigenin-7-O-glycoside, caffeic acid, chlorogenic acid, 3-coumaric acid, 4-coumaric acid, epicatechin gallate, ferulic acid, gallic acid, luteolin, luteolin-7-o-glycoside, naringenin-7-o-glycoside, narirutin, protocatechuic acid, pyrocatechol, pyrogallol, quinic acid, syringic acid, and vanillic acid. Notably, four of these such as chlorogenic acid, caffeic acid, syringic acid, and vanillic acid were the most abundant. Dandelion roots also contain a variety of compounds, including polysaccharides (inulin, mucilage, and pectin), carotenoids (lutein), fatty acids (e.g., myristic acid), sugars (glucose, fructose, and sucrose), essential minerals and vitamins [13].

Leaf extracts of dandelion prepared using different solvents (95% ethanol, 50% ethanol, and water) were analysed to determine the chemical composition of their phenolic acids [17]. Six phenolic acids were identified by high-performance liquid chromatography (HPLC), including chlorogenic, caffeic, p-coumaric, sinapic, ferulic, and chicoric acids. Among these, cichoric acid and sinapic acid were the most abundant across the various extracts of dandelion. The extract obtained using 50% ethanol exhibited the highest contents of cichoric and sinapic acids. Similarly, the presence of chicoric acid in extracts obtained from dandelion leaves was also confirmed [16]. The detailed analysis also showed the presence of other phenolic and flavonoid compounds, including caffeoyl diglucoside, 1-*o*-caffeoylquinic acid or 5-*o*-caffeoylquinic acid, 3-*o*-caffeoylquinic acid, caffeoyl glucoside, caffeoylmalic acid, luteolin diglycoside, luteolin-7-glycoside, dicaffeoylquinic acid, luteolin glycoside, and luteolin.

The extracts prepared from dandelion flowers, identifying several phenolic and flavonoid compounds have also been analysed [16]. These included caffeoyl diglucoside, 1-O-caffeoylquinic acid (or 5-O-caffeoylquinic acid), 3-O-caffeoylquinic acid, luteolin glycoside, luteolin diglycoside, chicoric acid, luteolin-7-O-glycoside, dicaffeoylquinic acid, luteolin, and chrysoeriol. Some of these compounds were also detected in the leaf extract. It was found that flowers are rich in phenols, alkaloids, flavonoids and saponins [18].

The phenolic acid and flavonoid contents extracted from dandelion root and herb juice have been studied [19]. The fractionation of the dandelion juice was done by solid-phase extraction. A total of 43 compounds were identified, including several quercetin glycosides and caffeoylquinic acids reported for the first time in dandelion, such as dicaffeoylquinic acids and chrysoeriol diglycoside. Among the bioactive compounds, monocaffeoylquinic acids were detected, including chlorogenic acid, cryptochlorogenic acid, and other quinic acid derivatives. Various dicaffeoylquinic acids were also characterized, such as 3,4-di-O-caffeoylquinic acid, 3,5-di-O-caffeoylquinic acid, and 4,5-di-O-caffeoylquinic acid. In addition, tartaric acid derivatives, including cis-caftaric acid, trans-caftaric acid, caftaric/coutaric acid derivative, trans-coutaric acid, and chicoric acid were identified, with chicoric acid being the most abundant compound in dandelion juice. The analysis also revealed the presence of hydroxycinnamic acid derivatives that do not contain a quinic acid group in their structure, such as caffeoyl hexosides, caffeoyl dihexoside, and other caffeic acid derivatives. Lastly, several flavonoids were found, including luteolin and chrysoeriol glycosides, as well as quercetin glycosides like quercetin triglycoside and quercetin pentoside.

A comprehensive chemical profiling of the dandelion plant was carried out using proton nuclear magnetic resonance (^1^H NMR) spectroscopy and gas chromatography–mass spectrometry [20]. The analysis revealed the presence of different amino acids in the whole plant, including alanine, 4-aminobutyrate, arginine, asparagine, carnitine, isoleucine, leucine, phenylalanine, proline, threonine, and valine. In addition to amino acids, several organic acids were identified, such as malic, acetic, citric, and succinic acids, along with glucose-1-phosphate, fructose, mannose, and inositol. The study also confirmed the presence of steroids and triterpenoids, notably sitosterol, stigmasterol, lanosterol, and β-amyrin. A wide range of fatty acids was detected, including hexadecanoic, heptadecanoic, octadecanoic, 9,12-octadecadienoic, linolenic, docosanoic, tricosanoic, tetracosanoic, and hexacosanoic acids. Additional bioactive constituents such as choline, O-phosphocholine, phytol, and tocopherol were also identified, further highlighting the phytochemical richness of dandelion.

The dandelion plant, both in its individual parts and as a whole, contains a diverse array of bioactive compounds. The major bioactive compounds, along with their chemical structures, were recently summarized [14]. Given their wide-ranging pharmacological properties and nutritional value, further research and valorisation of dandelion as a functional ingredient are warranted for both nutritional and therapeutic applications.

## 4. Applications and Benefits

All parts of the dandelion plant have demonstrated valuable properties and are widely used across various sectors, including the food, biomedical and pharmaceutical sectors.

### 4.1. Food Applications

Dandelion root has attracted considerable attention for its potential as a functional food, largely due to its high content of inulin-type fructans. These complex carbohydrates act as prebiotics, promoting gut health by inhibiting the growth of pathogenic bacteria in the gastrointestinal tract. Additionally, inulin-type fructans may contribute to the reduction in risks associated with cancer, obesity, and osteoporosis [21].

Leaves, flowers, and roots of dandelion are valued for their nutritional and functional properties and are increasingly explored for diverse food applications. Dandelion can be processed into a variety of products, including beverages such as tea, yogurt, functional drinks, and coffee. Moreover, it can be used for the preparation of such foods as biscuits, bread, pickles, and tofu. Roasted dandelion roots can also be drunk as coffee. This beverage is particularly recommended for individuals with diabetes, due to its content of fructo-oligosaccharides, which have demonstrated various health-promoting effects, including regulating blood glucose levels and reducing hyperglycaemia [13]. A study showed that it is possible to produce fermented compound wine from dandelion root [22]. Through a multi-stage production process involving enzymatic treatment, fermentation, and post-processing steps, a light-yellow dandelion-based wine was successfully developed. The technique included substrate hydration, enzymatic conversion using amylase and pectinase, controlled fermentation with yeast, and final stages such as filtration, aging, and sterilization. The resulting beverage exhibited a pleasant flavour profile and potential health-promoting properties.

The dietary effects of dandelion root extract on growth performance and disease resistance in rainbow trout (*Oncorhynchus mykiss*) have been investigated [23]. The study demonstrated that the extract exerted hypolipidemic and hypoglycemic effects while promoting muscle healing, improving biochemical blood parameters, and enhancing liver morphology in the fish.

Dandelion leaf extracts have been explored for their potential use as natural flavouring agents across a range of food products, including soft and alcoholic beverages, frozen dairy desserts, confectionery, baked goods, puddings, and cheeses [24]. Dandelion leaves are utilized in traditional dishes across various cultures. In Turkey, fresh leaves are often added as a seasoning to enhance the flavour of meals, while dried and ground leaves are used as a spice. In parts of Asia, young dandelion leaves are commonly incorporated into soups or stir-fried and served alongside brown rice [25]. Dandelion leaves are also consumed in salads [26]. Researchers also explored the development of a Chinese herbal salad using dried soil dandelion and hydroponic dandelion [27]. The first version of the salad was prepared using slices of dried soil-grown dandelion, whereas the second consisted of a vegetable-based salad topped with a sauce made from hydroponic dandelion powder, sunflower seeds, and olive oil. The results indicated that ≈47% of participants disliked the dried soil-grown dandelion, primarily due to its pronounced bitterness, which made it easily recognizable. In contrast, approximately 67% of participants expressed a preference for the hydroponic dandelion sauce. Overall, the taste acceptance rate for the dandelion-based salad exceeded 73%, indicating significant potential for its promotion and commercialization as a functional food with therapeutic benefits.

Dandelion flowers can be used to prepare syrups or to enhance the flavour of sweets such as candies, cakes, jellies, and puddings. In certain European regions, dandelion flower buds are pickled in vinegar and consumed similarly to capers, while they can also be added fresh to dishes such as pancakes and omelettes to enhance flavour [28]. In countries like Canada and the UK, whole dandelions are incorporated into beer recipes. In Belgium, the flowers are added to a type of beer known as saison, which is valued for its pronounced fruity aftertaste [25].

Dried and powdered dandelion flowers, leaves, and roots have been incorporated into various beverages and food products. For example, dandelion roots and leaves are commonly added to coffee and tea [29]. Several beverages were developed by combining dandelion roots with various types of Arabic and instant coffee, as well as incorporating dandelion leaves into tea preparations, either alone or blended with hibiscus. In total, six coffee-based drinks and five tea variants were formulated and subjected to sensory evaluation. The acceptance test results indicated that the blend of dandelion roots with light Arabic coffee and the hibiscus–dandelion leaf tea received the highest preference scores. These findings suggest that dandelion parts can serve as effective ingredients in the development of low-caffeine beverages with added health benefits, providing an affordable alternative rich in bioactive compounds for consumers seeking functional drinks. Additionally, another study investigated the effects of incorporating dandelion flower powder into wheat flour on dough properties and the quality of the resulting bread [30]. Wheat flour was partially replaced with dandelion flower powder at levels ranging from 1% to 6%, and the physical properties of both the dough and the resulting bread were evaluated. The incorporation of dandelion flower increased water absorption, dough development time, and dough stability. However, higher substitution levels resulted in a noticeable reduction in loaf volume and crumb brightness, accompanied by increased crumb hardness and yellowing, which negatively affected the overall sensory appeal. Despite these drawbacks, the addition of dandelion flower enhanced the nutritional profile of the bread by increasing its fibre, fat, and mineral content. Furthermore, it improved the bread’s antioxidant properties and elevated total phenolic content. These findings indicate that dandelion flower powder is a promising functional ingredient for bread, with a 2–3% substitution level providing an optimal balance between health benefits and product organoleptic quality.

Further research is necessary to explore the incorporation of dandelion extracts or dried forms into various food matrices, with the aim of improving consumer acceptance and maximizing the potential of this highly beneficial plant.

### 4.2. Biomedical and Dermatological Applications

Numerous studies and reviews have documented the health-promoting properties of dandelion. The plant has been reported to exhibit antioxidant, antibacterial, anti-inflammatory, antitumor, antiviral, hypoglycemic, and hypolipidemic activities, in addition to modulating hormone levels and protecting various visceral organs. The biomedical applications of dandelion extracts, principal pathways and molecules modulated by dandelion components in antitumoral, anti-inflammatory, antidiabetic, hepatoprotective, immunomodulatory, antimicrobial, and antioxidant activities were recently reviewed [14,31].

Its pharmacological profile encompasses anti-inflammatory, anti-rheumatic, antioxidant, anticancer, diuretic, choleretic, laxative, and hypoglycemic effects. Consequently, one of the primary applications of dandelion is in the development of therapeutic products, which can be formulated in diverse forms, including raw plant material, extracts, herbal infusions, and processed products such as powders, granules, tablets, and capsules. These products may be derived from the whole plant or from specific parts, such as roots, leaves, stems, or flowers [32].

Dandelion may play a role in the prevention or mitigation of complex diseases, including cancer, obesity, arthritis, hepatitis, and cardiovascular and gastrointestinal disorders [33]. For instance, sesquiterpene lactones exhibit anti-inflammatory and antibacterial activities. Phenolic acids and sesquiterpene lactones contribute to the plant’s antidiabetic potential. Sterols and triterpenes may help alleviate cardiovascular disorders. Coumarins demonstrate anti-inflammatory, bacteriostatic, anticoagulant, and anticancer effects. The different activities of dandelion plant leaf extracts were discussed, such as anti-inflammatory, anti-oxidant, neuro-protective, anti-cancer, immunostimulatory, anti-microbial, anti-diabetic, diuretic and kidney-protective activities. Dandelion leaves are commonly used as a diuretic and bitter digestive stimulant. Among medicinal plants, dandelion has attracted considerable attention owing to its potent antioxidant, anti-aging, antibacterial, and anti-inflammatory properties. Dandelion exhibits numerous medicinal properties, which are attributed to the bioactive phytochemicals (carotenoids, flavonoids, phenolic acids, polysaccharides, sesquiterpene lactones, sterols and triterpenes) present in its flowers, leaves, stems, and roots.

In traditional Indian medicine, boiled dandelion leaves were used as a remedy for bone fractures, and its roots were employed to manage liver and kidney disorders and to treat snakebites [14]. The dandelion flavonoids and their in vitro and in vivo antioxidant activities [34] and the phytochemical and pharmacological profile of dandelion have been recently reviewed [35]. Particularly, the dandelion sesquiterpenoids and their lactones, polyphenolic bioactive compounds, triterpenoids, flavonoids, sterols, volatile constituents, pigments, polysaccharide, and glycoside were analysed. The different effects, including antimicrobial, anti-inflammatory, anti-oxidative, anti-tumour, pharmacological and regulatory effect on lipid were discussed in detail.

Different approaches for green synthesis of nanoparticles using dandelion extract for biomedical applications have been reviewed [36]. Dandelion is a medicinal herb rich in bioactive phytochemicals and represents a promising natural template for the biosynthesis of metal and metal oxide nanoparticles, as well as nanoemulsions. The potential applications of dandelion extract–based nanomaterials for medicinal purposes have been explored, with particular emphasis on their antibacterial, antifungal, antiviral, and anticancer properties.

A detailed review on the biological and pharmacological characteristics of dandelions has been recently presented [14]. The potential medicinal applications of dandelion have been extensively investigated. Dandelion roots are primarily recognized for their digestive and hepatoprotective properties, while the leaves are commonly used as a diuretic and digestive stimulant. Moreover, the anti-inflammatory, antidiabetic, antitumor, and antioxidant activities of dandelion bioactive components have been widely documented.

The therapeutic potential of dandelion, together with its nutritional benefits, rich phytochemical profile, and efficacy in managing health conditions such as diabetes, inflammation, and cancer, has been extensively reviewed [37]. The antibacterial activity of dandelion extracts against two clinically relevant pathogens, *Escherichia coli* and *Staphylococcus aureus*, has been investigated [38]. Methanol, chloroform, and aqueous extracts of dandelion leaves and roots were prepared using Soxhlet extraction and maceration techniques. Among these, the methanol extracts demonstrated the highest antibacterial activity, which was primarily attributed to their content of flavonoids, tannins, and terpenoids.

The antioxidant, antimicrobial, and cytotoxic activities of dandelion root extract, along with its effects on breast cancer cells, have been investigated [39]. The phytochemicals identified in the extract, primarily taraxasterol, phenolic compounds, and triterpenoids, were found to induce cancer cell membrane disruption and cell cycle arrest through the activation of degradation pathways.

Dandelion has traditionally been used to treat various conditions, including skin infections, breast inflammation, lymphadenitis, conjunctivitis, sore throat, and lung abscesses [35]. Dandelion extracts have demonstrated potential in dermatological applications. A study reported that extracts from dandelion leaves and flowers offer significant photoprotective effects against Ultraviolet B (UVB)-induced damage in human dermal fibroblasts [40]. UVB light refers to ultraviolet radiation with wavelengths ranging from 290 to 320 nm, commonly known as the biological spectrum [41]. Specifically, the extracts suppressed the generation of reactive oxygen species and inhibited matrix metalloproteinase activity, both of which are associated with photoaging (sun damage) and cellular senescence (cessation of cell division). The protective effect was observed when the extracts were applied either shortly before or immediately after UVB exposure.

A comprehensive review on extraction, purification, structural features, biological activities, modifications, and applications in the fields of pharmaceutical and health food of bioactives from dandelion has been recently issued [42].

### 4.3. Limitations Associated with Dandelion Consumption

Adverse effects associated with dandelion consumption are relatively uncommon. Dandelion-based products have been granted “Generally Recognized as Safe” (GRAS) status by the U.S. Food and Drug Administration, allowing their use in dietary supplements [43]. Dandelion consumption is generally considered safe and well tolerated [33].

Despite its numerous benefits, dandelion presents certain limitations, such as the potential to trigger allergic reactions, accumulate environmental pollutants, exhibit a bitter taste, and show variability in phenolic composition. Additionally, its cultivation and harvesting may have agricultural impacts. Some consumers may also experience mild side effects, including diarrhoea, gastrointestinal discomfort, or skin irritation.

Allergic reactions to dandelion have also been documented. These reactions are primarily attributed to an 18 kDa Bet v 1–related protein iso-allergen present in its roots and stems. Sensitization has been observed in approximately 8.5% of patients with respiratory allergies, and anaphylactic reactions have been reported in atopic individuals following exposure to dandelion-containing pollen [44].

The common dandelion has been utilized as a bioindicator in passive biomonitoring studies to assess environmental contamination by elements such as Mn, Fe, Ni, Cu, Zn, Cd, and Pb [45,46]. These studies demonstrated that dandelions can accumulate heavy metals when grown near roads or in polluted soils. Therefore, for medicinal or culinary purposes, dandelion should be harvested from uncontaminated areas that are free from significant anthropogenic pollution.

Dandelion is characterized by a pronounced bitterness, which is mainly attributed to the presence of sesquiterpenoids compounds [24], specifically the eudesmanolide and germacranolide types unique to this plant [47]. Sensory evaluations have identified a bitter and grassy aftertaste. Notably, the intensity of the aftertaste increased with the content of dandelion flower [30].

Another limitation of dandelion is the variability of its phenolic composition [48]. The concentration of major phenolic compounds in dandelion varies across regions and is influenced by environmental factors such as temperature, precipitation, soil pH, and altitude. Consequently, the plant’s quality may fluctuate throughout the year, impacting its marketability. This underscores the importance of timely harvesting and proper storage practices to maintain the quality and consistency of dandelion products.

As a non-native and invasive species in regions such as Australia, North America, and South America, dandelion can significantly impact food and forage crop production. It competes with cultivated plants for essential resources, including nutrients, water, and light, often resulting in reduced agricultural yields [49].

## 5. Conventional Extraction Techniques

Conventional extraction techniques are based on different variants of solvent extraction techniques. The solvent extractions are very popular for extraction of different components from dandelions. For example, Soxhlet extraction technique allows the continuous recovery of analytes from a sample matrix using fresh solvent. They have long been employed for the recovery of bioactive compounds from plant materials. Several studies applied conventional approaches to investigate plant-based bioactives. For instance, the solid–liquid extraction of bioactive compounds from dried dandelion leaves using deionized water as the solvent was studied [50]. The process was conducted at controlled temperatures (40, 60, and 80 °C) for an extraction duration of 90 min. Among the tested kinetic models, Peleg’s model was identified as the most appropriate for describing the experimental data related to total polyphenol content and extraction yield. The highest extraction efficiency in terms of total polyphenols, antioxidant capacity, and overall yield was obtained at 80 °C.

The effects of extraction temperature on the structural characteristics and antioxidant activity of polysaccharides from dandelion leaves have also been evaluated [51]. In this study, dandelion leaf powder was dispersed in distilled water at a 1:20 (*w*/*v*) ratio and maintained either at 4 °C for 16 h or at 80 °C for 3 h. Although extraction efficiency increased at the higher temperature, a trend of structural degradation with increasing temperature was observed. The authors concluded that polysaccharides extracted at low temperature retain better structural integrity and can be used as antioxidants in food, medical, and biomaterial applications.

Dandelion leaves have been recognized as a rich source of polyphenols with strong antioxidant potential. A study demonstrated the plant’s potential as a valuable source for commercial applications [17]. The powdered dandelion leaves were extracted using three different solvents, ethanol-water (1:1 *v*/*v*), 96% ethanol, and water in a water bath at 80 °C. The results showed that the 50% ethanol extract of dandelion leaves had the highest amounts of total phenolic content, chicoric acid concentration, and antioxidant activity.

In addition to the aerial parts, several studies have also explored the extraction of bioactive compounds from dandelion roots which present significant potential as a commercial crop and an affordable natural source of inulin and antioxidants. The extraction of dry dandelion roots using a Soxhlet apparatus, highlighted the roots as a rich source of inulin-type fructans with varying chain lengths [52]. The research demonstrated that root extracts are effective as natural antioxidants and food additives, with the ability to enhance digestion and protect against diseases related to oxidative stress. Water extracts showed the highest inulin content (12% DW) along with significant levels of total phenolics. These water extracts also showed the strongest antioxidant activity positively correlated with phenolic content.

The polysaccharides from dandelion roots were extracted using a hot-water extraction technique and subsequently sulphated through the concentrated sulfuric acid technique [53]. Sulphation is a simple and effective technique for modifying the biological activity of polysaccharides by substituting their hydroxyl groups with sulphate groups. The resulting sulphated polysaccharides exhibited enhanced antioxidant and hypoglycemic activities, as well as improved value-added effects on probiotic growth, compared to the non-sulphated polysaccharides.

Polysaccharides were extracted from dandelion roots and leaves using water extraction followed by ethanol precipitation [54]. The findings provided valuable insights into the potential application of dandelion polysaccharides as natural nutritional agents for enhancing immune and antioxidant functions. It should be noted that ethanol is widely regarded as an effective and environmentally friendly solvent for the extraction of polyphenolic compounds from plant materials.

The aqueous extraction of polysaccharides from dandelion leaves at different temperatures (4 °C and 80 °C) has been performed [51]. The effects of extraction temperature on the structural characteristics and antioxidant activity of dandelion leaf polysaccharides were evaluated. The results demonstrated that extraction efficiency increased at higher temperatures, whereas the molecular weight showed a tendency to decrease with rising extraction temperature, indicating partial polysaccharide degradation.

The extraction, compositional analysis, and evaluation of the in vitro antioxidant and antiproliferative activities of dandelion seed oil have been investigated [55]. The extraction was performed using the petroleum ether reflux technique at 60–90 °C. The obtained dandelion seed oil exhibited strong antioxidant and antiproliferative activities, indicating its potential application as a natural antioxidant and in the development of health-promoting products.

The extraction technique has a significant influence on the structure and biological activity of the extracted compounds. Conventional extraction techniques have several limitations, including the risk of thermal degradation of polyphenols, high energy consumption and the use of large amounts of raw materials. These factors make the process expensive and less efficient [56]. More recent and sustainable extraction approaches focus on reducing energy consumption and minimizing the amount of plant material required. To improve extraction efficiency and lower environmental impact, various pretreatment techniques are now used.

The conventional approach typically involves hot-water extraction, a well-established, cost-effective, and environmentally friendly technique. However, this technique is characterized by low selectivity, high operating temperatures, and relatively low extraction efficiency, which may lead to structural degradation of the extracted molecules. Thermal extraction may have a negative influence on the phenolic compounds and enzymes activity.

## 6. Innovative Extraction Techniques

Applied extraction technique may have a significant influence on the structure and biological activity of the extracted compounds. To overcome the drawbacks of the conventional techniques, the innovative extraction techniques were developed. For dandelion the instant controlled pressure drop technique, supercritical and subcritical fluid-, ultrasound-, microwave-, enzyme-, and electrically–assisted extractions were tested.

### 6.1. Controlled Instantaneous Decompression

The controlled instantaneous decompression technique (DIC) was applied to extract the bioactive compounds such as phenols and flavonoids from dandelion roots and leaves [57]. DIC technique is based on a thermomechanical treatment. In this technique includes application of preliminary vacuum to eliminate air and ensure efficient contact between the saturated steam and the product, steam injection at high pressure for a short duration, controlled instant decompression for the instantaneous evaporation of internal water, and return to atmospheric pressure. The results showed that intermediate DIC conditions with pressure 0.25 MPa and time 20 s improved phenolic content (such as vanillic acid and hydroxycinnamic acid),and antioxidant activity compared to the control. The primary antioxidant catechin was the most abundant compound. These results suggest that DIC favours the release of phenolic compounds by promoting cell wall disruption and improving solvent accessibility, without inducing their degradation under moderate conditions. A key limitation of this process is the potential damage to heat-sensitive products during the initial pressure-increase phase [58].

### 6.2. Supercritical and Subcritical Fluid Extraction

Another innovative technique is the supercritical fluid (SupC) extraction, that combines high pressure with supercritical fluids like CO_2_ to destroy microorganisms without compromising the nutritional value or sensory qualities of the product [59]. In its supercritical state, CO_2_ exhibits the properties of both a gas and a liquid, enabling it to selectively dissolve target compounds while leaving no solvent residues. This technique represents a promising non-thermal alternative to traditional pasteurization, particularly for preserving bioactive compounds in food and pharmaceutical applications.

SupC fluid extraction has proven to be an effective technique for isolating bioactive compounds from natural sources, offering advantages such as shorter extraction times, reduced use of organic solvents, compatibility with thermo-sensitive compounds, and the ability to produce cleaner extracts. However, this technique also has certain limitations, including high initial equipment and infrastructure costs, the need for skilled operators, high energy consumption, and limited efficiency when extracting highly polar compounds.

The SupC fluid extraction with CO_2_ was used to extract triterpenoids and phytosterols from dandelion root and leaves [60]. The extracts obtained through this technique differ notably from those obtained by conventional extraction techniques in both appearance and chemical composition. Application of SupC fluid extraction allowed for the efficient and quantitative extraction of triterpenes and their esters, and produced higher concentrations of these compounds compared to extracts obtained by traditional Soxhlet solvent extraction technique.

A two-step SupC fluid extraction with CO_2_ combined with conventional extraction was employed for the valorisation of dandelion seeds [61]. It allows extraction of oils rich in essential linoleic fatty acids and pigments (chlorophylls and carotenoids). Moreover. the extracts showed promising antitumor. Various green extraction techniques, including SupC fluid extraction have been applied to enhance the recovery of bioactive compounds from both native and waste dandelion seeds [62]. The efficiency of combining environmentally friendly solvents, such as supercritical CO_2_ with absolute or aqueous ethanol, was also evaluated.

Subcritical (SubC) water extraction is also widely applied for the efficient recovery of bioactive compounds [63]. Water serves as a green, inexpensive, and readily available solvent in this technique. The process involves the use of pressurized water (typically above 5 MPa) at temperatures ranging from 100 °C to 374 °C. This environmentally friendly and cost-effective method offers higher extraction yields and improved quality compared to conventional techniques. Moreover, it enables the selective extraction of compounds with varying polarity, while offering advantages such as short extraction times, high efficiency, and relatively low instrumentation costs. However, certain drawbacks exist, including the risk of thermal degradation, possible formation of toxic residues, degradation of thermolabile compounds, increased energy consumption, and the need for additional post-processing steps such as drying or purification.

Dandelion leaf polysaccharides have been extracted using SubC water extraction at 120 °C [64]. Extracted complex polysaccharide was mainly composed of arabinose and galactose. Cells treated with this extract displayed obvious apoptotic morphology and this extract has potential as an anti-cancer agent.

The influence of temperature of SubC fluid extraction (110–160 °C) on polyphenols content and antioxidant activity has been studied [65]. It was demonstrated that dandelion flower extracts obtained at moderate temperatures (130–140 °C) with subcritical water have the highest polyphenol content and antioxidant activity.

### 6.3. Ultrasound-Assisted Extraction

Ultrasound-assisted extraction (UAE) utilizes ultrasonic wave energy to enhance the recovery of plant metabolites. Its mechanism involves the propagation of high-frequency sound waves through the extraction medium, leading to the formation and subsequent collapse of cavitation bubbles. This cavitation phenomenon generates localized high temperatures and pressures, resulting in the disruption of plant-cell structures and facilitating the release of intracellular bioactive compounds into the solvent [66]. UAE is an advanced extraction technique that offers several advantages, including high reproducibility, shorter extraction times, and lower energy consumption [67]. Ultrasound-assisted extraction technique enhances extraction efficiency while reducing or eliminating the need for toxic solvents. Moreover, the use of low extraction temperatures during the process help protect heat-sensitive bioactive compounds from degradation, contributing to a higher-quality extract [56]. However, this technique also presents certain limitations, such as the need for careful process optimization, the relatively high cost of equipment, potential degradation of soluble molecules under prolonged ultrasonic exposure, negative effects of pressure cavitation, and possible metal contamination from probe fragments.

UAE was performed using the powdered aerial parts of dandelion [68]. Extractions were conducted with ethanol and acetone solvents at 60 °C for 30 min. The acetone extract yielded the highest concentration of flavonoids. In contrast, the ethanol extract exhibited the strongest antioxidant activity, demonstrated by its highest radical scavenging activity and reducing power.

The relevance of UAE was also demonstrated in a study which examined the extraction of fresh and dried dandelion leaves, flowers, and roots for 15, 30, and 60 min using water, 40%, 70%, and 96% (*v*/*v*) water–ethanol mixtures as solvents [69]. The results demonstrated that the antioxidant activity of the extracts varied depending on the plant part, solvent composition, and extraction duration. The highest radical scavenging activity was observed in extracts from dried dandelion flowers obtained with 70% ethanol after 30 min, as well as in extracts from dried leaves using the same solvent after 60 min. Additionally, the antioxidant capacity of biological samples, measured by the Ferric Reducing Ability of Plasma (FRAP) assay was determined. This colorimetric method is based on a single-electron transfer mechanism. It indicated that extracts from dried leaves in 40% ethanol exhibited the greatest iron (Fe^3+^) reduction capacity, independent of extraction time. Overall, both fresh and dried leaf extracts had the strongest antioxidant potential, followed by flower extracts, with root extracts showing the lowest activity in most cases.

To further optimize UAE, the application of surface-active agents as alternatives to conventional organic solvents was investigated [16]. Micelle-mediated extraction offers a sustainable alternative by limiting the use of hazardous and volatile organic solvents. Dried powders of dandelion leaves and flowers were mixed with extraction solvent. A 2% (*v*/*v*) aqueous solution of Triton X-100 was used for micelle-mediated extraction of the plant materials, while 30% aqueous acetone served as the organic solvent control. Extractions were performed in an ultrasonic bath. The total phenolic content revealed that extracts obtained using water–organic solvent mixtures contained slightly higher phenolic levels compared to those from micellar extraction. Nonetheless, both extraction techniques yielded extracts with significant antioxidant activity. Quantitative analysis showed that acetone extracts of dandelion leaves contained the highest phenolic content, while Triton X-100 extracts of dandelion flowers also showed significant phenol levels. Among the plant parts studied, the roots contained the lowest levels of phytochemicals with antioxidant properties.

Ultrasound-assisted enzymatic extraction and characterization of polysaccharides from dandelion leaves has been studied [70]. The optimum parameters (ultrasound power, time, cellulase amount, and particle size) were determined. The effects of ultrasound-assisted enzymatic extraction on the antioxidant activity of dandelion polysaccharides have been investigated [71]. Optimal extraction parameters were established. The extracted polysaccharide, designated DANP-II, was found to consist primarily of glucose. DANP-II demonstrated notable antioxidant potential, suggesting its usefulness as a natural antioxidant for mitigating oxidative stress and providing theoretical support for the application of low–molecular-weight dandelion polysaccharides.

Ultrasound-assisted deep eutectic solvent extraction of chlorogenic acid from dandelion was studied at 70 °C [72]. The purified extract exhibited strong antioxidant activity and notable antibacterial efficacy. The effect of ultrasound pre-treatment of dandelion roots on the yield of hydroxycinnamic acids has been investigated [73]. Hydroxycinnamic acids, which are phenolic phytochemicals, include caffeic, ferulic, chlorogenic, isoferulic, and coumaric acids, compounds known for their high antioxidant capacity. The parameters of the pre-treatment process, as well as the technological conditions for producing tinctures from dandelion roots, were determined experimentally.

### 6.4. Microwave-Assisted Extraction

Among the innovative extraction techniques, microwave-assisted extraction (MAE) has gained significant attention as a green and efficient extraction technique. It uses (MAE) radiation to rapidly heat the solvent and sample matrix, thus enhancing heat and mass transfer processes [74]. The microwave-assisted extraction can not only effectively protect functional components, but also minimize solvent and energy consumption. Compared to conventional extraction techniques, microwave-assisted extraction provides several advantages, including shorter extraction times, lower solvent consumption, and enhanced process automation. By ensuring uniform heating of both the solvent and the sample matrix, (MAE) enhances extraction efficiency and yields a greater concentration of target compounds [75]. However, MAE also presents certain drawbacks, such as complex processing conditions, difficulty in reaction monitoring, high equipment costs, limited solvent compatibility, and challenges associated with scaling up to industrial applications.

The MAE technique was applied to extract polysaccharides from dandelion roots under optimized conditions (extraction time ≈ 40 min, temperature 80 °C, liquid-to-solid ratio 33:1, and microwave power 300 W) [76]. Under these parameters, the highest yield of dandelion root polysaccharides was obtained (≈25%). The findings suggest that dandelion roots represent a promising natural source of antioxidants with potential applications in food and pharmaceutical industries.

Microwave-assisted extraction of dandelion root polysaccharides has also been investigated [76]. The optimized extraction parameters (extraction time, extraction temperature, and solid–liquid ratio) were identified to maximize polysaccharide yield. The extracted polysaccharides were acidic in nature, with galactose and arabinose as the major constituents. The study concluded that acidic polysaccharides from dandelion roots represent a promising natural antioxidant source with potential applications in the food and pharmaceutical industries.

The UAE and MAE techniques were applied for extraction of inulin from dandelion roots [77]. Note that inulin is a polysaccharide that serves as a carbohydrate storage compound in plants. It is a water-soluble dietary fibre structurally similar to starch. The highest yield and degree of polymerization were obtained using UAE. These findings highlight the potential of dandelion-derived inulin as a functional ingredient for enhancing the taste and texture of food products, as well as a promising candidate for applications as a drug carrier or bio-based material. Furthermore, inulin has been reported to play an important role in the prevention and inhibition of cancer.

### 6.5. Electrically Assisted Extraction

In recent years, there has been growing interest in the use of electrically assisted non-thermal extraction techniques, which have minimal effects on nutritional components and help preserve the overall quality of products. Application of such innovative techniques, such as pulsed electric fields (PEF) and high-voltage electrical discharges (HVED) have demonstrated high effectiveness in extracting bioactive compounds from plant tissues [78,79].

The advantages of pulsed electric field (PEF)-assisted extraction include short processing times, reduced energy consumption, low operating and solvent costs, minimal environmental impact, and operation under mild temperature conditions [80,81]. This technique offers several advantages, including minimal cell wall disintegration, superior sensory properties, high retention of nutrients and vitamins, improved extraction yields, and the production of clean extracts with high purity and low coloration. It also facilitates the extraction of essential oils, proteins, pectin, and other valuable compounds. Additionally, PEF-assisted processing has been shown to enhance drying, vacuum freeze-drying, and freezing–thawing processes. Similarly, HVED technique disrupts cellular structures through electrical discharges, thereby improving the release of valuable phytochemicals such as phenolics and flavonoids [82]. Both PEF and HVED techniques have been successfully applied to various plant matrices, confirming their potential as energy-efficient, non-thermal alternatives for sustainable extraction processes. Disadvantages of these techniques are related with high capital cost of the equipment, which implies a high initial investment, possibility of dielectric ruptures in heterogeneous materials, restricted availability of commercial units, and negative effects related with electrochemical reactions at the electrode/medium interfaces, electrode corrosion and migrations of electrode materials into food systems.

However, to the best of our knowledge, there is a lack of studies on electrically assisted extraction from different parts of the dandelion. It would also be of interest to compare the efficiency of electrically-assisted extraction with that of other extraction techniques, as well as to explore the potential benefits of combining these techniques. The effects of different extraction techniques, including PEF treatment have been investigated [83]. Fresh, air-dried, and frozen samples were analysed using four different procedures PEF-assisted extraction, deionized water extraction, ethanol extraction, thermal treatment at 80 °C. The stability of polyphenols under UVB light exposure with biological spectrum for 28 days was also examined. Overall, the leaves exhibited the highest polyphenols content compared to the flowers, stalks, and roots. Extraction at 80 °C resulted in the greatest polyphenols yield compared with deionized water, ethanol, and pulsed electric field-assisted extractions.

Cold plasma treatment is a non-thermal, electrically assisted technique used for processing bioproducts at low temperatures [84]. This method employs charged and highly reactive gaseous species that interact with the components of biological materials. It is considered an environmentally friendly technology due to its minimal water requirements and ability to selectively modify biomass components. Cold plasma treatment also offers potential for in-line integration, high efficiency, and cost-effectiveness. However, as a relatively new technology, it still requires process optimization and currently faces limitations in large-scale industrial application. Additional drawbacks include the potential generation of corrosive by-products, high energy consumption, and challenges related to water use and system maintenance.

The dandelion roots were subjected to dielectric barrier discharge cold plasma treatment [85,86]. The plasma was generated using a high-voltage direct current power supply (40 kV, 56 kHz, 10 mA) connected to a dielectric barrier discharge reactor. The plasma discharge occurred between two electrodes separated by dielectric barriers. Subsequently, aqueous infusions were prepared, and changes in colour, total phenolic content, antioxidant activity, and sensory attributes were evaluated. The results indicated that the effects of this pretreatment on the quality characteristics of dandelion root infusions varied with exposure time and particle size. Overall, these findings suggest that cold plasma pretreatment has the potential to improve the quality and antioxidant properties of dandelion root infusions.

Key characteristics of the innovative extraction techniques are presented in Table 2, including their efficiency, selectivity, operating conditions, and scalability.

## 7. Conclusions

Various techniques for extracting bioactive compounds from dandelion are currently under intensive development. Both conventional and emerging methods are employed, including instant controlled pressure drop, supercritical and subcritical fluid extraction, ultrasound-assisted extraction, microwave-assisted extraction, and electrically assisted extraction techniques. All parts of the dandelion plant have demonstrated valuable properties and are widely utilized across multiple sectors, including food, biomedical, and pharmaceutical industries. The application of these techniques to different plant parts (flowers, stems, roots, and leaves) may result in varying extraction efficiencies for bioactive compounds. Despite its numerous benefits, dandelion presents certain limitations, such as the potential to trigger allergic reactions, accumulate environmental pollutants, exhibit a bitter taste, and show variability in phenolic composition. Some consumers may also experience mild side effects, including diarrhoea, gastrointestinal discomfort, or skin irritation. Each extraction technique is associated with specific advantages and limitations, and particularly, studies on electrically assisted extraction of bioactive compounds from dandelion remain limited. Addressing these challenges through further research and optimization could enhance the practical application and economic viability of innovative extraction techniques in industrial biomass conversion processes. Further research is also needed to compare the efficiency and selectivity of these emerging techniques, to optimize process parameters, and to explore the full spectrum of bioactive compounds that can be extracted from dandelion. Investigating the integration of different emerging technologies to achieve synergistic effects could improve biomass pretreatment and extraction efficiency, paving the way for more sustainable and effective industrial applications. Addressing scale-up challenges, such as energy use, process stability, and cost-effectiveness, will be essential for industrial application. In addition, the implementation of standardized quality control measures will help ensure consistent bioactive compound profiles and minimize safety risks.

## Figures and Tables

**Table 1:** Distribution of major bioactive compounds in dandelion roots, leaves, and flowers [15,16,17].

Roots	Leaves	Flowers
3-Coumaric acid 4-Coumaric (p-coumaric) acid Apigenin-7-O-glycoside Caffeicacid Chlorogenicacid Epicatechin gallate Ferulicacid Gallicacid Luteolin Luteolin-7-O-glycoside Naringenin-7-O-glycoside Narirutin Protocatechuicacid Pyrocatechol Pyrogallol Syringicacid Vanillicacid	1-O- or 5-O-caffeoylquinic acid 3-O-caffeoylquinic acid 4-Coumaric (p-coumaric) acid Caffeoyl diglucoside Caffeoyl glucoside Caffeoylmalic acid Chichoricacid Ferulicacid Luteolin Luteolin diglycoside Luteolin glycoside Luteolin-7-O-glycoside	1-O- or 5-O-caffeoylquinic acid 3-O-caffeoylquinic acid Caffeoyl diglucoside Chichoricacid Chrysoeriol Dicaffeoylquinic acids (3,4-, 3,5-, 4,5-diCQA) Luteolin Luteolin diglycoside Luteolin glycoside Luteolin-7-O-glycoside

**Table 2:** Comparison of innovative extraction techniques: efficiency, selectivity, operating conditions, and scalability.

Extraction Technique	Efficiency	Selectivity	Operating Conditions	Scalability	Reference
Controlled Instantaneous Decompression (DIC)	High	High	High pressure and temperature, rapid decompression	Laboratory and pilot scale	[87]
Supercritical Fluid Extraction (SFE)	Limited efficiency when extracting highly polar compounds	High	Moderate temperature	Industrial	[88]
Subcritical Water Extraction	High	High	High temperature & pressure	Laboratory and pilot scale	[89]
Ultrasound-Assisted Extraction (UAE)	Moderate	Moderate	High frequency sound waves, low–moderate temperature	Laboratory & industrial scale	[90]
Microwave-Assisted Extraction (MAE)	High	Moderate	Microwave radiation, moderate temperature	Laboratory & industrial scale	[91]
Pulsed Electric Fields (PEF)	Moderate-High	High	Short high-voltage pulses, moderate temperature	Pilot and industrial scale	[92]
High-Voltage Electrical Discharges (HVED)	High	Moderate	Electrical discharges, low temperature	Laboratory and pilot scale	[93]
Cold Plasma Treatment	High	High	Low temperature	limitations in large-scale industrial application	[84]

## Data Availability

No new data were created or analyzed in this study. Data sharing is not applicable to this article.

## Abbreviations

DIC	Controlled instantaneous decompression
HVED	High-voltage electrical discharge
MAE	Microwave-Assisted Extraction
PEF	Pulsed electric field
SubC	Subcritical
SupC	Supercritical
UPLC-MS	Ultra-Performance Liquid Chromatography–Mass Spectrometry
UAE	Ultrasound-Assisted Extraction
UVB	Ultraviolet B
